# Microthrombi and hypercoagulability’s contribution to delayed cerebral ischemia after aneurysmal subarachnoid hemorrhage

**DOI:** 10.1177/0271678X261463964

**Published:** 2026-06-17

**Authors:** William W Wroe, Hussein A Zeineddine, Spiros L Blackburn, Jaroslaw Aronowski, Devin W McBride

**Affiliations:** 1 The Vivian L Smith Department of Neurosurgery, McGovern Medical School, The University of Texas Health Science Center at Houston, Houston, TX, USA; 2 Department of Neurology, McGovern Medical School, The University of Texas Health Science Center at Houston, Houston, TX, USA

**Keywords:** Platelets, subarachnoid hemorrhage, delayed cerebral ischemia, delayed neurological deficits, vasospasm, microthrombi, DND, DCI

## Abstract

Despite increasing knowledge over the last several decades of the factors contributing to delayed cerebral ischemia (DCI), it remains a significant cause of morbidity after aneurysmal subarachnoid hemorrhage (aSAH). The hypercoagulable state induced by aneurysm rupture is critical to most proposed mechanisms of DCI either through its direct contribution to microthrombi formation, or through the indirect effects of the products of coagulation on vasospasm, and inflammation. In this review we summarize clinical evidence of hypercoagulability after aSAH as well as both the clinical and pre-clinical evidence of its role in DCI, highlighting the role that microthrombi and NETosis are thought to play.

## Introduction

Aneurysmal subarachnoid hemorrhage (aSAH) has an estimated incidence of 8/100,000 globally,^1^ but has a devastating impact on the overall burden of stroke in the population due to the high morbidity and the relatively young age of those afflicted.^2^ Of the patients who survive to have their aneurysms treated, delayed cerebral ischemia (DCI) is the leading cause of morbidity and mortality.^3^ DCI has an incidence of roughly 30% and typically occurs 3–14 days after aSAH often leading to infarction or delayed neurological deficits (DND).^4^ It is defined clinically as either the development of a focal neurologic deficit, a decline in the Glasgow Comma Score of 2 or more points, or the presence of infarction arising withing 6 weeks after aSAH that cannot be attributed to alternative causes.^5^ In addition to the classically described culprit, large artery vasospasm, it is now known that the causes of DCI are multifactorial, and include microthrombi, cortical spreading ischemia, microvascular constriction, and inflammation.^6^ Despite these many factors contributing to DCI, it is not clear how important each phenomenon is nor how independent each are.

There is strong evidence that aSAH patients become hypercoagulable soon after ictus,^7^ and it is intuitive, if not yet proven, that this hypercoagulability can lead to the phenomenon of microthrombosis seen in autopsy studies of these patients.^8–10^ Although less intuitive, there is evidence that increased coagulation contributes to several of the other factors thought to cause DCI such as microvascular dysfunction^11–13^ and inflammation.^14–16^

In this narrative review, we examine the clinical and preclinical evidence demonstrating the role that hypercoagulability, microthrombi, and immuno-thrombosis play after aSAH. In particular we will highlight the recent experimental evidence regarding NET and microthrombi formation after aSAH, as well as what the growing clinical use of dual antiplatelet therapy (DAPT) and other antithrombotics after aSAH means regarding the potential efficacy of antiplatelets and antithrombotics in preventing DCI.

## Methods

This paper was created as narrative review of the topic. From the search engines PubMed and Google Scholar, the following keywords and their combinations were searched: delayed cerebral ischemia, vasospasm, aneurysm rupture, subarachnoid hemorrhage, hypercoagulability, coagulation, microthrombi, platelets, platelet function, von Willebrand factor, thrombo-inflammation, immuno-thrombosis, and NETosis. Identified abstracts were screened for relevance. Additional relevant references were identified from the bibliography of extracted articles. Due to the narrative review design, the content may be subject to selection bias.

Regarding the experimental data discussed below, there are several different models used to induce SAH in rodents, each with their strengths and limitations. Overall, blood injection models control SAH volume very well but tend to cause less severe SAH whereas perforation models cause variable SAH volumes but are more severe. Reviews specifically detailing differences can be read elsewhere.^17^

## Hypercoagulability after SAH

There exists strong clinical evidence that a hypercoagulable state exists soon after aneurysm rupture. Tjerkstra et al. reviewed 19 studies evaluating thromboelastography (TEG) and rotational thromboelastometry (ROTEM) parameters and showed hypercoagulable profiles in aSAH patients compared to controls. Additionally, increased hypercoagulability was associated with multiple adverse events including DCI, deep vein thrombosis, and poor outcome at both hospital discharge and outpatient follow-up.^7^ In a later prospective study, Tjerkstra et al. validated a significant correlation between hypercoagulable parameters and both poor outcome and DCI.^18^ While the association between hypercoagulability and poor outcome has been confirmed by other groups,^19–21^ a recent prospective study by Raatikainen et al. was unable to find an association between elevated ROTEM values and DCI.^20^

Several mechanistic factors have been proposed as contributing to the hypercoagulability after SAH including increased platelet function and aggregation,^22,23^ an imbalance between von Willebrand factor (vWF) and ADAMTS13 activity,^24,25^ increased fibrin formation and decreased fibrinolysis,^26^ and thrombo-inflammation.^15^ The evidence implicating these factors, and the strategies used to ameliorate them, will be discussed in the following sections after an initial examination of the most obvious end product of hypercoagulability, microthrombi.

## Microthrombi after SAH

### Evidence of microthrombi and clustering

While cerebral thrombi had been observed within cortical arteries,^27^ large artery thrombi have not been considered as major drivers of DCI.^28,29^ The first report of cerebral microthrombi after SAH was the autopsy study by Suzuki et al. which showed clear evidence of microthrombi in the brain of a patient with symptomatic vasospasm. The authors noted that microthrombi consisted of platelets and multi-nuclear leukocytes.^30^ Since this was a single patient study, a correlation between microthrombi and DCI could not be assessed until their case series of six aSAH patients, four of who had developed symptomatic vasospasm, including the patient that was presented in their initial case report. Thirteen regions of the brains were examined for presence of microthrombi. Taken together, the microthrombi count was significantly elevated in the patients with symptomatic vasospasm (microthrombi count range for symptomatic vasospasm was 180–699 versus 15–17 for non-symptomatic vasospasm). This was the first study to investigate if, and identify that, cerebral microthrombi positively correlated with DCI in humans. Also of note, while the composition of the microthrombi varied between patients, platelets, fibrin, and leukocytes were present.^8^ Two decades later, an autopsy case series of 29 patients by Stein et al. evaluated microthrombi after SAH and found that microthrombi burden followed a timeline suggesting it to be a potential cause of DCI and histological ischemia.^10^ While this study confirms the presence of microthrombi and provides further evidence of a positive correlation between microthrombi and DCI, a potentially very important observation was that “the sites . . . at which clusters of microthrombi are most numerous correspond to the sites of maximal [neuronal necrosis] and infarction.”^10^ This observation is interesting since one would not expect DCI to occur if microthrombi were randomly dispersed throughout the microvasculature, unless there was an extremely high burden. However, clustering of microthrombi could cause local damage via reduced perfusion and ischemia. The only other evidence of clustering is from our lab using a mouse model of SAH where we have observed and quantified clusters of microvascular occlusions (either by microthrombi or intravascular neutrophil extracellular traps (iNETs)) that localize with infarcts and positively correlated with the development of DND in SAH mice.^31,32^ While the evidence from the autopsy studies could not provide information regarding causality of DCI by microthrombi, our recent mouse studies suggest brain microvessel occlusion (by either microthrombi or iNETs), and in particular their clustering are causes of DCI in SAH mice.^31,32^

### Timeline of microthrombi formation

**Figure 1.**
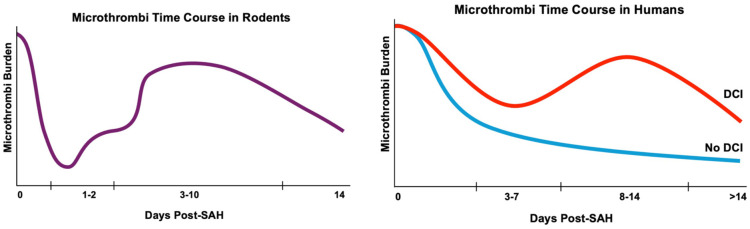
Proposed microthrombi burden over time: (left) rodent microthrombi burden with an initial peak immediately after SAH, and then a second peak during the DCI window. Rodent data is extrapolated from Friedrich et al.,^11^ Sehba et al.,^33^ El Amki et al.,^35^ Pisapia et al.,^36^ and Dienel et al.^38^ and (right) human microthrombi burden over time showing that patients with DCI have a similar second peak during the DCI window as the proposed rodent timeline. Humans that did not suffer DCI do not have this second peak. Human data is extrapolated from Stein et al.

In addition to the human autopsy studies, there is extensive preclinical evidence of the pathological nature of microthrombi after SAH. Microthrombi have been detected in the cerebral microvasculature of mice as early 10 min after SAH induction,^11,12,33^ and these microthrombi block flow leading to reduced microvasculature perfusion.^11^ Mechanistically important is the finding that these microthrombi are strongly associated both spatially and temporally with vasoconstriction in arterioles.^11,12^ Moreover, the degree of vessel constriction positively correlates with microthrombi, and vessels that had no blood flow always contained microthrombi.^12^ This immediate formation of microthrombi is intuitive since it is a compensatory mechanism to the blood vessel rupture causing SAH. Sehba and Friedrich also reported that microthrombi count peaked at 10 min and again at 24 h after SAH, before no longer being present by 48 h.^34^ In contrast, El Amki et al. and Pisapi et al. both found near sham levels of microthrombi on day 1 post-SAH with microthrombi count peaking between days 2 and 5 before decreasing (although still significantly elevated compared to sham).^35,36^ El Amki et al. continued their time course study out to 14 days post-SAH and observed a second peak on day 10 with a significant decrease on day 14 (although microthrombi count was significantly higher than sham at all time-points after day 1).^35^ SAH causing significant burden of cerebral microthrombi on days 7 or 8 is supported by two other groups.^37,38^ Despite minor discrepancies in the cerebral microthrombi time course, possibly due to differing SAH models, SAH severity, or varying immunohistochemical techniques, when taken together, the timeline of cerebral microthrombi burden in these five studies suggests two peaks, one very early on and a second during the DCI time window which is very similar to the timeline of microthrombi accumulation reported for humans (Figure 1).^10^

The bimodal pattern for microthrombi formation after aSAH had been proposed by Stein et al. The authors speculated that the early increase in microthrombi is caused by aSAH (presumably the aneurysm rupture) followed by a second increase when thromboembolism and thrombolysis are competing.^10^ As shown in experimental models, SAH causes rapid microthrombi formation, as early as 10 min after SAH,^11,12,33^ and this early microthrombi formation coincides with early hypercoagulability reported in aSAH patients.^7^ In a recent paper, we measured four factors of platelet activation in an animal model of SAH and observed a bimodal pattern in two of the factors (platelet activating factor and thromboxane B2) with an early peak at day 1 and a second peak on day 5; this data indicates that there is initial and a delayed activation of platelets.^32^ Regarding the second peak of microthrombi observed in humans and animal models, thromboembolism and thrombolysis very likely play roles and are both competing. As described within, vWF is increased, promoting clot formation, while fibrinolysis is decreased, allowing for clots to persist. Based on experimental evidence (i.e. NETs formation pattern, and NETs colocalizing with microthrombi)^31,39^ and clinical data (i.e. markers of NETs),^31,40^ we propose that the second peak has thrombo-inflammation and immune–thrombosis components as contributing factors.

It must be noted however that the human data supporting this second peak phenomenon comes from a single study containing only 29 patients and so must be interpreted with caution.^10^ However, as currently the only way to detect cerebral microthrombi is on post-mortem autopsies; and as both the morality rate after aSAH^41^ and the general practice of autopsies after disease related deaths has reduced^42^ significantly since this cohort (1983–1995), it is unlikely that a larger study will be produced until an in-vivo imaging technique that can evaluate for microthrombi is developed.

### Pathological nature of microthrombi

Multiple studies have examined the relationship between microthrombi and adverse neurological and histological outcomes using animal models of SAH. Within the first few hours after SAH, both microthrombi count and location correlate with arteriole constriction and reduced perfusion.^11,12,34^ Sabri et al. found that on day 2 post-SAH, not only did microthrombi count positively correlated with neuronal cell death, but more importantly, there was spatial correlation,^13^ suggesting a casual role of microthrombi in neuronal cell death. A separate study reported that, on day 10 after SAH, both cortical microthrombi count and apoptosis were elevated. However, the study did not test for a direct correlation between the outcomes.^35^ Using a rabbit cisterna magna shunt SAH model, Andereggen et al. did not observe a correlation between microthrombi count neuronal cell death on day 1^43^; the lack of correlation in the rabbit model may be due to the time point that was studied (day 1), small sample size, or the difference in SAH induction as this is the only animal model that utilized the cisterna magna shunt. Only one study has tested for and reported a positive correlation between microthrombi and infarction, with the studied time point being 7 days after SAH.^38^ Since infarction is a hallmark of DCI, this study provides strong evidence of a possible causal link between microthrombi and DCI.

Another key pathophysiological event after SAH is vasospasm and microvessel constriction. Wang et al., in a very interesting experiment, placed CSF from human aSAH patients onto the cortical surface of rats and found that CSF from DCI patients caused worse microvascular constriction in the rats compared to aSAH patients without DCI. The authors did not analyze microthrombi in this arm of the experiment, but in a separate arm they showed that 24 h after SAH by cisternal injection, rats showed both increased microvascular constriction and capillary microthrombosis, although no attempt to establish co-localization was made.^44^ A recent study by our lab found that SAH caused both elevated microthrombi and elevated microvessel constriction, however, again no correlation was tested for.^45^ Three studies have observed spatial correlation between microthrombi and microvessel constrictions, but all three studies evaluated time points within 72 h of SAH,^11,12,33^ so additional studies are needed to determine if microthrombi and microvessel constriction correlate during the DCI time window. Additionally, no study has provided evidence regarding which event (microthrombi formation or microvessel constriction) precedes the other.

## Platelet counts, activation, and hyperactivity

Multiple clinical studies have evaluated platelet function and found that it is both increased after aSAH and associated with poor outcomes.^46–48^ More specifically platelet activation and function has been found to correlate with DCI and DND.^47,49–52^ Frontera et al. showed that patients who suffered DCI had elevated max amplitude on TEG, a marker of platelet function, when compared to patients not experiencing DCI.^49^ Although this marker of platelet function may not be robust enough to be widely used as a biomarker for DCI risk.^21^ Elevated makers of platelet activity such as platelet derived growth factor (PDGF),^53^ mean platelet volume,^52,54^ thromboxane,^50,51^ platelet activating factor,^55^ platelet aggregation,^50,51^ and coated platelets^56^ have also all been associated with DCI or symptomatic vasospasm in aSAH patients. Decreased platelet counts in the days following aSAH have also been correlated with DCI, suggesting a possible pathway where DCI caused by microthrombi formation leads to a relative mild consumptive thrombocytopenia.^57,58^ Since this is speculative, experiments are needed to verify that mild consumptive thrombocytopenia is the result of microthrombi deposition in the brain.

While clinical evidence of platelet function and factors suggests platelets may be important in SAH pathogenesis, including DCI, preclinical data shows that platelet hyperactivity causes microthrombi formation and deposition in the brain vasculature. Immediately after SAH, platelets can be seen adhering and aggregating in the cerebral microvasculature,^11^ leading to a reduction in cerebral blood flow and a concomitant reduction of platelet velocity.^59^ This reduced platelet velocity exacerbates aggregation leading to a self-reinforcing, pro-thrombotic cycle. Moreover, leukocytes also have increased adhesion and reduced velocity, and early after SAH, platelet–leukocyte “plugs” can be seen.^59,60^ Sabri et al. reported that the cell adhesion molecule P-selectin, a marker of platelet activation, may contribute to the increased “stickiness” of platelets. Their study demonstrated that P-selectin was expressed only in the microvessels of SAH mice and not sham mice, and that P-selectin co-localized with microthrombi.^13^ This mechanism is further supported since pre-treatment with a P-selectin antibody reduced rolling and adherent platelets to sham levels along with near-complete elimination of platelet–leukocyte adhesion; this suggests that elevated P-selectin levels cause microvessel occlusion via platelet–leukocyte plugs. Ultimately, three studies by two different labs have reported that a P-selectin antibody is beneficial for SAH rodents through normalized platelet and leukocyte velocity within the microcirculation.^13,59,60^ However, it remains to be determined if a P-selectin antibody improves neurobehavioral outcomes or prevent DCI. Clinical evidence also suggests that P-selectin might be important; Frijns et al. found that aSAH patients who developed DCI had significantly higher P-selectin levels (at the onset of DCI) compared to aSAH patients not developing DCI.^61^

As previously discussed, there is a clear relationship between microthrombi formation and microvascular constriction.^11–13,44^ Whether constriction or thrombosis comes first is unknown, but they are likely involved in a self-reinforcing cycle. The molecular drivers behind this cycle remain unknown, however preclinical evidence implicates decreased NO^13^ and pericyte PDGF expression.^62^

Large vessel, or angiographic vasospasm, has also been attributed to increased platelet activity. Hasegawa et al. showed that aSAH patients with worsening vasospasm had a higher percentage of activated platelets and elevated TEG values. While the evidence is correlative, the authors then showed that platelet aggregation and activation peaked 3–6 days after SAH in dogs, which corresponds with the timeline of vasospasm and DCI.^63^ Direct evidence of platelets causing vasoconstriction was provided by an ex-vivo study of the rabbit basilar artery by Tanaka et al.^64^ Two days post-SAH via cisterna magna injection or sham surgery, the authors harvested the basilar artery from rabbits and placed the arteries in a solution with a varying amount of platelets. Arteries from sham and SAH rabbits showed a dose response with increasing tension as platelet count increased, but the arteries taken from SAH animals showed a significantly increased vasoconstrictive response compared to sham. Additionally, this platelet-induced tension could be reduced by pre-treatment with an antiplatelet drug: indomethacin (a cyclo-oxygenase inhibitor), dazoxiben (a thromboxane synthetase inhibitor), or ONO-3708 (a selective thromboxane A2 antagonist).^64^ Satoh et al. demonstrated that platelet rich plasma (PRP) alone injected into the cisterna magna of dogs causes angiographic vasospasm of the basilar artery both at 1 h and 7 days. At the 1-h time-point, PRP induced a similar degree of constriction to that of whole blood. However, at 7 days post-injection, the whole blood group displayed more severe vasospasm then the PRP group.^65^

The mechanisms behind the large vessel constriction caused by platelets have been further investigated in correlational studies. Juvela et al. reported that increased thromboxane release between days 5 and 14 was associated with worsening radiographic vasospasm and DCI in patients after aSAH, but platelet aggregation was unchanged.^51^ Alksne and Branson induced SAH in macaque monkeys and found that the locations of angiographic vasospasm, evaluated by pre-sacrifice cerebral angiography, correlated with significant endothelial damage and platelet aggregation 4–14 days post-SAH.^66^

Another platelet activator, ADP has also been tested in animals, but due to difficulty in detecting it, ADP has not been evaluated following SAH in patients. Ohkuma et al., using felines, demonstrated that intra-arterial ADP, a potent platelet activator, caused constriction in the basilar artery in both the sham and SAH groups. Despite sham animals displaying a constricted basilar artery, there was no platelet aggregation in the constricted basilar artery, whereas in the SAH group there was a large number of adherent platelets on days 4 and 7. Despite significant platelet adhesion on days 4 and 7, there were no adherent platelets 1 h after SAH, and only a few adherent platelets at both 2 and 14 days.^67^ This timing corresponds well with vasospasm and DCI.

In addition to platelet activators, platelets release numerous molecules through degranulation which may propagate other pathophysiological mechanisms. An example of this is PDGF for which there is a significant body of literature in animal models showing that PDGF is elevated after SAH,^68^ administration of PDGF causes large vessel vasoconstriction,^69–71^ and subsequently PDGF inhibition after SAH reduces vasoconstriction.^69,70^ This literature is best summarized in a review article by Ghali et al.^72^

A final, although much less studied, potential mechanism for platelets role in DCI is through cortical spreading depolarization or ischemia.^73^ Microthrombi^74^ and microvascular constriction^75^ have both been associated with cortical spreading depolarization either through their causative role in ischemia, or in the case of microthrombi, through platelets induction of glutamate meditated neuronal toxicity.^74^ Glutamate is known to be elevated in patients after aSAH^76,77^ and can cause spreading depolarizations and ischemia.^78^

## Platelet inhibition

Due to the long-suspected connection between platelet induced hypercoagulability and DCI, there have been numerous clinical studies evaluating the effect of antiplatelet drugs (Table 1). An early study by Juvela et al. suggested that patients taking aspirin prior to aSAH who had elevated salicylate levels in their urine (proving recent intake) had reduced incidence of DCI.^79^ Subsequent retrospective studies have not substantiated this finding,^80–85^ however none of the newer studies confirmed therapeutic levels of aspirin at the time of aSAH leading to the possibility that their statistical power is diluted due to non-therapeutic patients being included in the treatment arm.

**Table 1.** Clinical antiplatelet trials cited in this study divided by target.

Antiplatelet target	Author	Study design	Investigation	*n* ^1^	DCI or sVS	Radiographic vasospasm	Hemorrhagic complications
COX inhibition	Hop (2000)	RCT	ASA vs placebo after aSAH	50	No difference	N/A	No difference
	Van Den Bergh (2006)	RCT	ASA vs placebo after aSAH	161	No difference	N/A	N/A
	Juvela (1995)	RR	ASA and NSAID use before and after aSAH	291	Reduced risk of DCI with pre-hospital ASA use. No difference with in hospital use	N/A	N/A
	Gross (2014)	RR	ASA use at time of aSAH	274	No difference	No difference	N/A
	Enriquez-Marulanda (2019)	RR	Antiplatelet use (>95% ASA) at time of aSAH	267	No difference	N/A	N/A
	Al-Mufti (2021)	RR	ASA use at time of aSAH	186	No difference	No difference	N/A
	Toussaint (2004)	RR	ASA use at time of aSAH	305	Decreased permanent disability from sVS w/ ASA use (23% v 50%; *p* = 0.069)	N/A	Increased aneurysm re-bleed w/ ASA use
	Bruder (2018)	RR w/ PSM	ASA use at time of aSAH	288	No difference	No difference	No difference
	Sebok (2022)	RR	ASA use at time of aSAH	1033	No difference	N/A	No difference
P2Y12	Nagahama (2018)	RR	DAPT (clopidogrel + ASA) vs no AP medications after aSAH	161	Reduction in DCI w/ DAPT (2% vs 22%; *p* = 0.001)	Reduction in rVS w/ DAPT (13% vs 33%; *p* = 0.003)	No difference
	Darkwah Oppong (2019)	RR	DAPT (clopidogrel + ASA) vs ASA monotherapy vs no AP medication	580	No difference between DAPT and ASA monotherapy	No difference between DAPT and ASA monotherapy	Increased rate w/ DAPT vs ASA monotherapy.
	Wallace (2020)	RR	DAPT (clopidogrel + ASA) vs ASA monotherapy after aSAH	142	No difference	No difference	No difference
	Sun (2020)	RR	DAPT (clopidogrel + ASA) vs no AP medications after aSAH	166	Reduction in DCI w/ DAPT (1.5% vs 10.9%; *p* = 0.03), reduction in sVS w/ DAPT (1.5% vs 12.9%; *p* = 0.01)	N/A	No difference
	Ditz (2021)	RR	DAPT (clopidogrel + ASA) vs no AP medications after aSAH	160	No difference	No difference	No difference
	Ma (2025)	RR	DAPT (clopidogrel + ASA) vs no AP medications after aSAH	259*	No difference	Reduction in mod-severe rVS w/ clopidogrel + ASA (32% vs 56%; *p* < 0.001)	No difference
	Ma (2025)	RR	DAPT (ticagrelor + ASA) vs no AP medications after aSAH	229**	No difference	No difference	No difference
Thromboxane A2 synthase	Yano (1993)	NRT	TA2 synthetase inhibitor vs standard of care after aSAH	28	Increase in DCI w/ synthetase inhibitors (62% vs 13% *p* < 0.05)	N/A	N/A
	Suzuki (1989)	RCT	TA2 synthetase inhibitor vs placebo after aSAH	285	Reduction in DCI w/ TA2 synthetase inhibitor (10% vs 33%; *p* < 0.01)	Reduction in mod-severe rVS w/ TA2 synthetase inhibitor (41% vs 63%; *p* < 0.05)	No difference
	Tokiyoshi (1991)	RCT	TA2 synthetase inhibitor vs standard of care after aSAH	24	Reduction in sVS w/ TA2 synthetase inhibitors (38% vs 73%; *p* < 0.005)	N/A	No difference
Phosphodiesterase type 3	Suzuki (2019)	RR w/ PSM	Cilostazol at varying doses vs standard of care after aSAH	156	Reduction in DCI w/ cilostazol 300 mg/day (4% vs 33 %; *p* < 0.005)	No difference	No difference
	Suzuki (2011)	RCT	Cilostazol vs placebo after aSAH	100	No difference	No difference	No difference
	Senbokuya (2013)	RCT	Cilostazol vs placebo after aSAH	109	Reduction in DCI w/ cilostazol (11% vs 29%; *p* = 0.030)	Reduction in rVS w/ cilostazol (50% vs 77%; *p* = 0.006)	No difference
	Matsuda (2016)	RCT	Cilostazol vs placebo after aSAH	148	Reduction in sVS w/ cilostazol (11% vs 24%, *p* = 0.031), No difference in infarction	No difference	No difference
	Sugimoto (2018)	RCT	Cilostazol vs placebo after aSAH	50	Reduction in DCI w/ cilostazol (13% vs 40%; *p* = 0.036)	No difference	No difference
GP IIb/IIIa	Zanty (2020)	RCT	Tirofiban vs placebo after aSAH	30	Reduction in DCI w/ tirofiban (6% vs 33%; *p* = 0.04)	Reduction in rVS w/ tirofiban (6% vs 42%; *p* = 0.01)	No difference

RCT: randomized control trial; NRT: non-randomized trial; RR: retrospective review; PSM: propensity score matching; DCI: delayed cerebral ischemia; sVS: symptomatic vasospasm; rVS: radiographic vasospasm; ASA: aspirin; aSAH: aneurysmal subarachnoid hemorrhage; DAPT: dual antiplatelet therapy; AP: antiplatelet.

1 Study (n) includes both treatment and control group.

* Apirin and clopidogrel group and control group. **Aspirin and Ticagrelor group and control group.

The role of post-rupture antiplatelet use has been evaluated in several randomized controlled trials (RCTs). Hop et al. and Van den Bergh evaluated aspirin after SAH in relatively small trials of 50 and 161 patients, respectively, with neither study observing a measurable effect on DCI or vasospasm.^86,87^ Thus, aspirin has not been widely used for preventing DCI alone, although more recent studies have tested DAPT with aspirin which is discussed later.

Thromboxane A2 synthetase inhibitors were evaluated in three Japanese trials between 1980 and 1990.^88–90^ As thromboxane A2 is downstream of arachidonic acid metabolism by COX-1 (the target of aspirin), one would think aspirin and thromboxane A2 synthetase inhibitors would either both work or neither would work. However, COX-1 leads to products that can inhibit platelets in addition to products that promote platelet activation and aggregation. So, targeting thromboxane A2 synthetase inhibitors would affect only the platelet-inhibitory COX-1 products. The largest study, which was conducted by Yano et al. with 285 patients in a RCT, reported a significant reduction in radiographic vasospasm and delayed infarcts.^88^ The other two smaller studies enrolled only 24^90^ and 28^89^ patients and showed mixed results, with Tokiyoshi et al. suggesting benefit while Yano et al. suggested no efficacy with thromboxane A2 synthase inhibitors.^89,90^ Despite positive findings in two studies, including the moderately sized trial by Yano et al., there have been no subsequent studies in humans or animals evaluating thromboxane A2 synthetase inhibitors after SAH.

Cilostazol is a phosphodiesterase III inhibitor used most frequently for peripheral vascular disease for its antiplatelet and vasodilatory effects. Cilostazol was first evaluated in SAH animal models by multiple Japanese research groups, and was found to be effective in reducing radiographic vasospasm and endothelial damage.^91–93^ There are several proposed mechanisms that are thought to contribute to the observed improvement including decreased platelet–leukocyte interaction,^94^ increased NO production,^95^ and PDGF inhibition.^96^ Cilostazol has since been extensively studied in aSAH patients, including several RCTs.^97–100^ Multiple meta-analyses of these trials, plus other non-randomized cohorts, have been done, the largest totaling over 6000 patients,^101^ with all showing an efficacious role of cilostazol in the treatment of DCI, as well as radiographic vasospasm.^101–103^ The most recent cilostazol study conducted was a retrospective analysis published in 2019 indicating that a dose of 300 mg/day was more effective than 200 mg/day used in all the prior RCTs suggesting an even greater effect in preventing DCI and vasospasm than what had been shown previously.^104^ Due this growing evidence of efficacy, cilostazol is now used routinely in Japanese hospitals for the treatment of DCI, often in combination with either fasudil or clazosentan.^105,106^ A recent clinical trial examined combination of cilostazol and nimodipine treatment in the United States; unfortunately the trial was terminated early due to poor enrollment (NCT04148105).^107^

The most recent single antiplatelet drug to have been tested as part of a RCT for SAH was a 7-day infusion of tirofiban, a platelet aggregation antagonist targeting glycoprotein IIb/IIIa. This Phase 1/2a study, labeled iSPASM, enrolled only 30 patients, but treatment was associated with a reduction in DCI and radiographic vasospasm in the 18 patients receiving tirofiban.^108^ This same group retrospectively reviewed their institutions experience in treating ruptured aneurysms with tirofiban after stent assisted coiling, and found a significant reduction in radiographic vasospasm and a trend toward reduction in DCI when compared to those who underwent coil embolization without any antiplatelets.^109^ The effects of tirofiban need to be evaluated in a large, multi-center clinical trial which is pending (NCT07493577).

Since there are an increasing number of ruptured aneurysms being treated with endovascular modalities necessitating antiplatelet regimens, several clinical studies have now evaluated DAPT and its effects on DCI. Two early retrospective studies by Darkwah Oppong et al. and Wallace et al. included 43 and 19 SAH patients, respectively, started on DAPT after aneurysm treatment. Neither study found any reduction in DCI or vasospasm with DAPT when compared to SAH patients treated with either aspirin alone or no antiplatelet medications.^110,111^ However, several moderately-sized retrospective studies have found reductions in either DCI or vasospasm with DAPT treatment.^112–115^ The two largest of these studies, by Nagaham et al. and Ma et al. had 85 and 82 DPAT treated patients, respectively.^112,115^ No meta-analysis has been conducted yet that includes the more recently published DAPT cohorts, but the totality of current studies suggests an association between DAPT and reduced DCI or vasospasm, although findings are inconsistent and confounded by evolving endovascular practices. One addition to this conclusion is that aside from a small subset of patients in the study by Ma et al. who received ticagrelor, all patients treated with DAPT had aspirin and clopidogrel as their two antiplatelet drugs. Thus, it is possible that there are other efficacious combinations of DAPT which have not been examined that included drugs such as cilostazol or tirofiban.

A Cochrane review published in 2007 evaluated the role of antiplatelets after SAH and determined that antiplatelet treatment to prevent DCI was not recommend.^116^ There are several caveats to this conclusion though, with the primary one being that, due to the small sample sizes in most studies, only four trials met their standard for “good quality” studies. Additionally, results from trials using various antiplatelet drugs were grouped together for an overall conclusion about antiplatelet drugs as a whole. This point is relevant since not all antiplatelet drugs are equal and there are potential differences in what receptor is targeted for platelet inhibition. Lastly, the Cochrane review was completed before the majority of the cilostazol trials or the frequent use of DAPT after endovascular treatment of ruptured aneurysm. More recent meta-analyses including the cilostazol studies and some of the DAPT studies do suggest a potential benefit for antiplatelet treatment of SAH.^117–119^

The major argument against using antiplatelets in SAH patients is the risk of hemorrhagic complications; however, the meta-analyses analyzing antiplatelet use after aSAH do not show any increase in hemorrhagic complications with either single or dual antiplatelet use. These studies combined all types of hemorrhagic complications such as aneurysm re-rupture, intracranial hemorrhage, and other hemorrhages related to the various invasive procedures that patients hospitalized after aneurysm rupture undergo.^117–119^ What is likely most illuminating to the risks of antiplatelets in aSAH patients are those studies evaluating intracranial hemorrhages after external ventricular drain placement (EVD), a common procedure performed after aSAH to treat hydrocephalus. The studies by Nagaham et al. and Ma et al. examined this in their cohort of patients started on DAPT after endovascular treatment of ruptured aneurysms and found no significant increase in the rates of EVD-related intracranial hemorrhages compared to those not on any antiplatelets.^112,115^ This suggests that treatment with antiplatelets would be safe to administer if they can be proven to be efficacious in treating DCI.

## Von Willebrand factor and ADAMTS13

vWF is a glycoprotein present in the blood plasma that induces platelet adhesion and aggregation. It is initially secreted as ultra-large vWF multimers (ULvWF) from platelet-derived alpha-granules and endothelium-derived Weibel–Palade bodies in response to vascular injury or stress. Under normal physiologic circumstances, ULvWF is cleaved into its less thrombogenic version vWF by ADAMTS13.^120^ In the context of SAH, patients develop elevated levels of vWF and lower levels of ADAMT13 activity,^121^ which likely contributes to the persistent thrombogenic state during the DCI time window, the development of DCI, and increased mortality.^24,55,122^

The known relationship between vWF activity and platelet-induced thrombosis makes vWF and ADAMTS13 attractive targets for reducing microthrombi formation and subsequent DCI, however there have been no human trials to date using drugs targeting either factor. Despite no clinical evidence, multiple animal studies have looked at vWF activity after SAH and demonstrated that inhibiting vWF improves neurological outcome.^25,123,124^ Vergouwen et al. showed that wild-type mice treated with recombinant ADAMTS13 (rADAMTS13) had both reduced microthrombi formation and less brain injury, as measured by microglial activation, 48 h after prechiasmatic blood injection SAH. Additionally, they showed that ADAMTS13^−/−^ mice with SAH, compared to wild-type SAH mice, had increased microthrombi and brain injury and that rADAMTS13 treatment reversed this deleterious outcome in the ADAMTS13^−/−^ mice.^124^ Muroi et al. confirmed that rADAMTS13 reduced microthrombi and cell death in SAH mice, but also reported that rADAMTS13 improved neurobehavior scores at 24 and 48 h.^123^ Wan et al. evaluated VWF^−/−^ mice and showed that, consistent with the theory that microthrombi are detrimental after SAH, there was decreased neuronal injury in the vWF^−/−^ mice compared to wild-type and ADAMTS13^−/−^ mice.^25^ The fact that they did not find a find a significant amount of microthrombi 2 h after SAH, the timepoint for this study, in any arms of their experiment is consistent with Sehba et al.’s prior study that demonstrated a reduction in microthrombi between the peaks at 10 min and 24 h.^33^

While these studies highlight vWF and ADAMTS13 as potential therapeutic targets, these three preclinical studies only evaluated outcomes during the EBI period. However, it seems likely that the reduction in microthrombi, and any subsequent reduced ischemia, would persist throughout the DCI window. The most intuitive way that reduced vWF activity can prevent microthrombi is via decreased platelet aggregation and fibrin formation. However, vWF also has roles in leukocyte function as vWF bound to endothelial cells promotes leukocyte adherence, rolling,^125^ and extravasation out of the microvasculature.^126^ vWF is also known to contribute to the formation of neutrophil extracellular traps (NETs), a major contributor to thrombo-inflammation.^127^ While immuno-thrombi are not the focus of this review, vWF contributing to both NETs and microthrombi (both critical pathophysiological events caused by SAH^31^) may mean that vWF is a key protein promoting both thrombo-inflammation and immuno-thrombosis.

## The coagulation cascade and fibrinolysis

The coagulation cascade ends with the conversion of fibrinogen to fibrin and the formation of a stable fibrin clot that forms the “glue” between platelets and the damaged vessel. Autopsy studies and preclinical studies provide evidence that microthrombi, thought to contribute to DCI, are partially composed of fibrin.^8,10^ In addition to clot-stabilization, several products of the coagulation cascade are known to induce vascular constriction.^128^ Because of these deleterious effects the coagulation cascade and its end product fibrin are potential targets to inhibit in order to reduce DCI.

Several clinical trials have found that markers of increased fibrin production and decreased fibrinolysis are associated with cerebral vasospasm after SAH. Kasuya et al. identified that increased fibrinopeptide levels were present in CSF in those patients with poor CSF blood clearance. These patients subsequently developed delayed infarcts more often than patients with less fibrinopeptide and more rapid blood clearance.^129^ Suzuki et al. found that CSF levels of thrombin–antithrombin III complex (TAT) and prothrombin fragments, both markers of coagulation system activation and elevated risk for thrombosis,^130^ were higher in aSAH patients with vasospasm than those without vasospasm on days 7 through 9.^131^ Ikeda et al. and Ji et al. reported that TAT as well as plasminogen activin inhibitor (PAI-1), a molecule that inhibits fibrinolysis, were also elevated in the CSF^132^ and blood,^133^ respectively, in patients with vasospasm throughout the DCI window, and lower in those with good clinical outcomes.^132,133^ Interestingly, specific gain-of-function genetic variations of the SERPINE1 gene (which encodes for PAI-1) have also been associated with DCI and poor functional outcome after aSAH in humans, potentially due to decreased fibrinolysis.^134^

Some additional insight into the roles that fibrin and fibrinolysis play in DCI can be gleaned from the effects of routine treatments. Antifibrinolytics, such as tranexamic acid (TXA), were once frequently given after aneurysm rupture in order to prevent re-bleeding prior to surgical fixation. Although TXA has been proven to reduce the incidence of re-bleeding, it is no longer recommended for routine use partly due to the increased risk of developing DCI.^135^ Nimodipine, a vasodilating dihydropyridine calcium antagonist, is also routinely given to aSAH patients due to its proven efficacy in reducing DCI.^136^ The mechanism behind its utility in aSAH though is unclear as it does not significantly change the rate of angiographic vasospasm. One potential mechanism for nimodipine is by augmenting fibrinolysis; Roos et al. showed that aSAH patients treated with nimodipine had both significantly increased fibrinolytic activity and significantly decreased plasma PAI-1 levels compared to untreated patients.^137^ Nimodipine’s fibrinolytic activity has been demonstrated in other clinical scenarios as well.^138^ This suggests that increased fibrinolysis, and not vasodilation, may be the explanation behind nimodipine’s effectiveness for reducing DCI incidence. And while no studies have been done to date directly examining nimodipine’s effect on microthrombi formation, Schwarting et al. demonstrated a reduction in microvessel vasospasm after SAH with nimodipine treatment in a murine endovascular perforation model,^139^ and as previously described there is a positive correlation between microvessel spasm and microthrombi.^11,12,33^

Only a few preclinical studies have looked into the use of fibrinolytics and anticoagulants in SAH models. Tsurutani et al. injected antithrombin III (an endogenous inhibitor of coagulation) into the CSF of rats priors to SAH induction and observed lower levels of D-dimer (a marker of fibrinolysis) within the CSF which coincided with reduced basilar artery vasospasm on day 4, the only time-point evaluated.^140^ This agrees with the later discovery by Maeda et al. that, in ex-vivo rabbit basilar arteries, contractile response to thrombin was increased in those arteries that had been pre-exposed to SAH. This increased reactivity to thrombin was reduced by heparinizing the autologous blood before injection.^141^ It is Maeda et al.’s hypothesis that this increased contractile response was due to activation of proteinase-activated receptors-1 (PAR-1) since PAR-1 is upregulated after SAH, and that heparin administration worked by preventing the activation of thrombin and its subsequent activation of the thrombin-PAR-1 pathway.^142^ Intravenous (IV) heparin infusion was shown in a mouse blood injection model to reduce neuroinflammation and apoptosis.^143^ The only SAH animal study to look at the administration of a fibrinolytic, in this case urokinase-type plasminogen activator (uPA), was done by Pisapia et al.; a single dose of uPA 3 h after SAH induction in mice via endovascular perforation significantly reduced brain microthrombi formation to near sham levels 48 h after induction. No neurological or ischemic injury evaluation was performed in this study.^36^

There have been no clinical trials evaluating IV administration of fibrinolytics but there have been several trials examining anticoagulants. In a small study of 24 patients on either direct oral anticoagulants or vitamin K antagonists prior to aneurysm rupture there was no statistical difference in the rate of DCI when compared to matched controls.^144^ Three total retrospective studies have evaluated IV heparin treatment after SAH. Two of the studies, both from the same group, evaluated a low-dose IV regimen, up to 10 U/kg/h, that was administered in patients who otherwise did not have an indication for anticoagulation, in the hopes of reducing DCI and vasospasm.^145,146^ The third study evaluated outcomes in aSAH patients who were started on therapeutic anticoagulation for well-established reasons, such as pulmonary emboli or deep vein thrombosis.^147^ All three studies showed significantly reduced incidence of either DND, DCI, or vasospasm in those treated with IV heparin versus control, and a meta-analysis of all three studies (with a total of 895 patients) supported the use of IV heparin.^148^

To date there have been two RCTs evaluating anticoagulation for the treatment of DCI, both using enoxaparin (low molecular weight heparin). Siironen et al. randomized 170 patients to either placebo or 40 mg of subcutaneous enoxaparin daily and found no difference in DCI or long term clinical outcome.^149^ Contrarily, Wurm et al., randomizing 120 patients to either 20 mg of daily subcutaneous enoxaparin or placebo, observed that enoxaparin lead to a reduction in vasospasm, an improvement in long term outcome, and a marked reduction of delayed infarctions (3.5% for enoxaparin vs 28.3% for placebo, *p* < 0.001).^150^ The discrepancies between these two trials are difficult to reconcile, but it should be noted that they are both decades old and used low dose regimens consistent with “prophylactic” and not “therapeutic” levels of the drugs. At most centers in the current era, patients routinely receive similar doses of either unfractionated or low molecular weight heparin for deep vein thrombosis prevention once the ruptured aneurysm has been secured and the rates of DCI remain ~30%. A RCT of “therapeutic” dosing of nadroparin, a low molecular weight heparin similar to enoxaparin, has recently been proposed which will hopefully shed light onto the matter.^151^

## Immuno-thrombosis

The innate immune system has an important procoagulant role in many different disease states, and interactions between immune cells and the traditional players in the coagulation cascade occur at many points along the chain of events leading to the final fibrin-platelet plug.^14^ Pattern recognition receptors, expressed by many innate immune cells, recognize coagulation factors (e.g. Factor Xa and thrombin) and trigger leukocyte activation. vWF (mentioned previously for its role in coagulation) can induce leukocyte tethering, rolling,^125^ extravasation,^126^ and may also trigger the formation of intravascular NETs, promoting immuno-thrombosis.^127^

As it is well-established that aneurysm rupture leads to both a profound inflammatory^152,153^ and hypercoagulable response, it is a natural line of investigation as to whether disrupting inflammation can prevent microthrombosis. It has been shown by Ishikawa et al. that 60 min after endovascular perforation in mice, cerebral flow velocities in both platelets and leukocytes are significantly reduced, and that platelet–leukocyte “plugs” form, thereby reducing blood flow in cerebral capillaries. As previously discussed, treatment with a P-selectin inhibitor reversed this phenomenon.^59,60^ A recent study by Croci et al. showed that tocilizumab, an antagonist of the pro-inflammatory cytokine receptor IL-6, not only reduced radiographic vasospasm in rabbits subjected to SAH, but tocilizumab also reduced microthrombi formation and neuronal cell death, suggesting a deleterious role for immuno-thrombosis.^154^

Multiple experimental studies have reported that neutrophil depletion in SAH mice reduces DCI,^31,155,156^ which has been suggested to be by reduced microvascular occlusion^157^ or intravascular NET (iNET) formation.^158^ Supporting the latter, studies focusing only on the inhibition of NETosis, and not neutrophils directly, have shown a reduction in both EBI^159^ and DCI.^31^ Multiple groups have also evaluated the therapeutic potential of NETs degradation after SAH using either DNase I or RNase I, with promising results in the EBI window.^39,159,160^ Important to understanding the underlying mechanism of efficacy, Hao et al. demonstrated that NETs colocalize with microthrombi, and that pre-treatment with either a neutrophil depleting antibody or DNase I treatment 1 h after SAH significantly decreased both the presence of NETs and microthrombi 24 h after SAH. Both treatments also concomitantly ameliorated neurological decline, neuronal damage, and cerebral edema at 24 h, suggesting a possible role immuno-thrombosis during EBI.^39^

Despite these promising results in the EBI time frame, the only two studies to evaluate iNETs degradation in the DCI window (both using intraperitoneal injection of DNase I) did not show any decrease in microvessel constriction at 5 days,^161^ or day 7 neurological decline and infarct volume.^31^ Nakagawa et al. did an additional experiment using DNase I, injecting it intrathecally, and found a reduction in microvessel constriction on day 5 after SAH. Neither neurobehavioral testing or infarct analysis were performed.^161^

**Figure 2.**
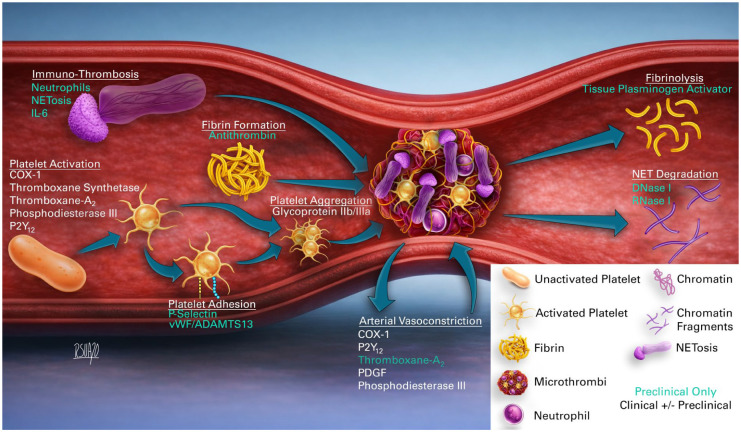
Steps involved in microthrombi formation after SAH and molecular targets that have been examined in both SAH patients and preclinical models. SAH induced microthrombi is proposed to be formed by both traditional components of coagulation such as fibrin and platelets, but also by products of NETosis. Illustration created using Adobe Illustrator.

In further support of immune–thrombosis and thrombo-inflammation, and in agreement with colocalization of NETs and microthrombi after SAH, our lab has reported that SAH leads to brain microvessel occlusion by both iNETs and microthrombi, and that preventing either microthrombi or iNETs causes a concomitant reduction in the other.^31,32^ Figure 2 shows a schematic of the potential interplay between microthrombi and NETosis, as well as what molecular targets have been investigated to interrupt this mechanism. Although this relationship has not been proven yet after human aSAH, given that leukocytes are known to be present in human microthrombi,^8^ it is plausible and supports the idea that thrombo-inflammation and immuno-thrombosis are key drivers of DCI and poor outcome after SAH.

In support of the pre-clinical work on thrombo-inflammation, multiple clinical studies have attempted to track NETosis after aSAH using various markers such as myeloperoxidase (MPO), citrullinated histone H3 (CitH3), and neutrophil elastase (NE), among others.^31,40,159,162^ The studies have shown significant elevations in many of these NETosis markers after aSAH when compared to controls. What is more interesting is the finding by Schneider et al. and Zeineddine et al. that during the DCI window certain markers, CitH3 and NE, respectively, were significantly more elevated in aSAH patients that went on to develop DCI versus those that did not develop DCI.^31,40^

This is an exciting finding which calls for a clinical trial, however several translational road blocks exist. Systemic neutrophil depletion is not a realistic clinical option, and the breakdown of NETs after they have formed using RNase or DNase has had at best mixed results in mouse models.^31,161^ Additionally, the most common drugs used to prevent NETosis in murine studies inhibit PAD4, a target for which there is no FDA approved drug. Overcoming these hurdles would require either substantial investment to obtain new FDA approval, or the repurposing of already approved drugs that have the coincidental “off-target” effect of NETosis inhibition such brensocatib,^163^ disulfiram,^164^ and colchicine among others,^165^ none of which have been examined in pre-clinical aSAH models.^158^

**Figure 3.**
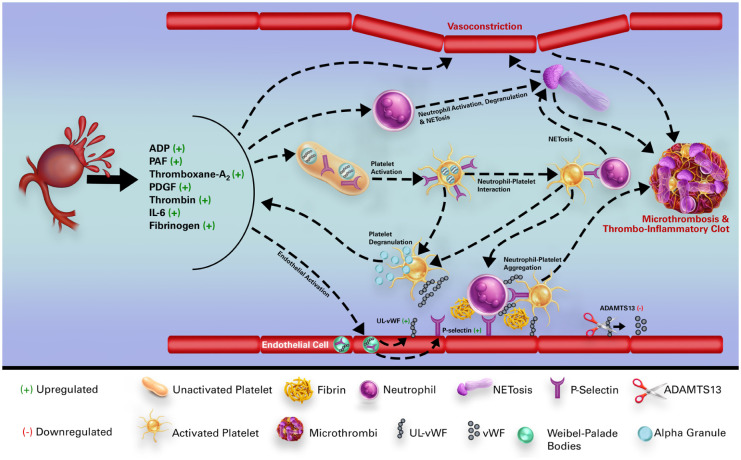
There are extensive feedback loops between coagulation factors and the immune system that are initiated after aneurysm rupture. It is proposed that the downstream effects of these interactions lead to the pathological outcomes of vasoconstriction, thrombo-inflammatory clots, and microthrombi formation. Illustration created using Adobe Illustrator.

An additional feature that has not been evaluated in humans is whether the various anti-thrombotics that have been tested in humans have led to any reduction in NETosis markers. As illustrated in Figure 3, there is extensive crosstalk between pro-thrombotic and pro-inflammatory factors with many positive feedback loops, and it is plausible that a reduction in platelet function, fibrin formation, or other pro-thrombotic factors could result in a decrease in NETosis.

## Conclusion

aSAH is known to induce a systemic state of hypercoagulability which may be due to hyperactivity of coagulation, platelets, or reduced activity of fibrinolysis. The final product of this hypercoagulability in the brain is the phenomenon of microthrombi which likely contributes to DCI in a variety of ways, including promoting focal ischemia. Despite limited reports, both clinical and preclinical studies suggest that microthrombi clusters might be a primary mechanism by which microthrombi cause poor outcome, but this needs to be further investigated.

Multiple preclinical trials have shown various drugs to be efficacious in the reduction of microthrombi and DCI, but as of yet no clinical trial of antithrombotics has demonstrated clear efficacy to be considered a standard treatment for DCI. Several candidates though, such as cilostazol, and tirofiban have shown promise and we await the results of the ongoing iSPASM2 (NCT07493577) and CASH trial (NCT07144956) examining tirofiban and cilostazol, respectively. And although a randomized control trial involving DAPT is unlikely, as endovascular treatment of ruptured aneurysms with either flow diversion or stent assisted coiling gains in popularity, the pool of retrospective data will increase, allowing for a more precise estimation of DAPT’s effect on DCI.

The synergy between the innate immune system and coagulation is also likely crucial in the pathology of DCI and further investigation into how treatment of one affects the other is needed. In particular the inhibition of NETosis has shown significant efficacy in murine models, but as of yet no clinical trial targeting NETosis has been attempted. As of now there are no FDA approved PAD4 inhibitors, the most common target used in pre-clinical models, but a current clinical trial is under way in the US in patients with rheumatoid arthritis (NCT06103877) offering some hope of FDA approval in the future. Additionally, pre-clinical studies should be run examining the effect of drugs with known “off target” inhibition of NETosis to see if human trials are warranted.
